# Integrin α2 gene polymorphism is a risk factor of coronary artery lesions in Chinese children with Kawasaki disease

**DOI:** 10.1186/s12969-021-00494-5

**Published:** 2021-02-08

**Authors:** Jia Yuan, Zhiyong Jiang, Meiai Li, Wei Li, Xueping Gu, Zhouping Wang, Lei Pi, Yufen Xu, Huazhong Zhou, Baidu Zhang, Qiulian Deng, Yanfei Wang, Ping Huang, Li Zhang, Xiaoqiong Gu

**Affiliations:** 1 Department of Cardiology, Guangzhou Women and Children’s Medical Center, Guangzhou Medical University, Guangzhou, 510623 Guangdong China; 2 Department of Blood Transfusion and Clinical Lab, Guangzhou Institute of Pediatrics, Guangzhou Women and Children’s Medical Center, Guangzhou Medical University, Guangzhou, 510623 Guangdong China; 3 Department of Clinical Lab, Guangzhou Institute of Pediatrics, Guangzhou Women and Children’s Medical Center, Guangzhou Medical University, Guangzhou, 510623 Guangdong China; 4 Department of Clinical Biological Resource Bank, Guangzhou Institute of Pediatrics, Guangzhou Women and Children’s Medical Center, Guangzhou Medical University, Guangzhou, 510623 Guangdong China; 5 Department of Blood Transfusion , Clinical Biological Resource Bank and Clinical Lab, Guangzhou Institute of Pediatrics, Guangzhou Women and Children’s Medical Center, Guangzhou Medical University, Guangzhou, 510623 Guangdong China

**Keywords:** Kawasaki disease, ITGA2, Genetic susceptibility, Polymorphism

## Abstract

**Background:**

Kawasaki disease (KD) is a systemic vasculitis, and the formation of coronary artery lesions(CAL) is its most common sequela. Both genetic and environmental factors are considered to be important factors of in KD. Integrin α2 (ITGA2) is a transmembrane receptor that is associated with susceptibility to several diseases, but its relevance to KD with CAL is unclear.

**Methods:**

We genotyped ITGA2 rs1126643 in 785 KD patients with the CAL and no-CAL(NCAL) (300 patients with CAL, and 485 age- and sex-matched patients with NCAL). OR (95% CI) and adjusted OR (95% CI) were used to evaluate the intensity of the association.

**Results:**

We found a significantly increased risk of KD with CAL associated with ITGA2 rs1126643 genotypes (CT vs CC: adjusted OR = 1.57, 95% CI = 1.16–2.12, *P* = 0.0032; CT/TT vs CC: adjusted OR = 1.49, 95% CI = 1.12–2.00, *P* = 0.0068; T vs C: adjusted OR = 1.66, 95% CI = 1.16–2.51, *P* = 0.0165). Moreover, we found that carriers of the CT/TT genotype had a significant risk of KD with coronary artery lesion susceptibility for children ≤60 months of age, and the CT/TT genotype was significantly associated with an increased risk of SCAL formation and MCAL formation when compared with the CC genotype.

**Conclusion:**

ITGA2 rs1126643 was associated with increased susceptibility and severity of CAL in KD.

## Background

Kawasaki disease (KD) is a systemic vasculitis first described by Dr. Kawasaki in 1974 in Japan [1]. It primality affects children below 5 years old, especially in Asian countries. The incidence of KD is highest in Japan, Korea and Taiwan ranging from 66/100,000 to 234/100,000 in children younger than 5 years old [2, 3]. The exact etiology of KD is not known [4]. Both genetic and environmental factors have been reported to play important role in KD [5]. The most common sequel of KD is coronary artery lesions (CAL), which is known to predominantly occur in young children (84% ~ 86% of all cases occur in children between 6 months and 5 years of age) with a male predominance (approximately 1.5 ~ 1.8 times higher than females) [6, 7]. CAL included coronary artery dilation, aneurysms, or fistula formation. All patients were identified only by echocardiography.

Antiplatelet therapy is routinely applied in KD treatment. Elevated platelet counts often develop in the acute phase of KD. This causes hyperplatelet function in which platelets become adherent, deformed, aggregates and release various cytokines and inflammatory factors. As a consequence, blood hypercoagulates causing vascular endothelial injury, collagen exposure, and triggers chemotaxis of various cytokines, all of which results in the formation of vascular inflammatory lesions [8]. Low-dose aspirin was recommended to prevent platelet activation and aggregation. All the patients received low doses of aspirin(3–5 mg/kg/day).Integrin α2 (ITGA2), which encodes the alpha subunit of the transmembrane receptor for collagens and related proteins, is located on chromosome 5q23–31 [9]. The encoded protein forms a heterodimer with a beta subunit responsible for adhesion of platelets and other cell types to the extracellular matrix. KD is defined as a vascular injury disease similar to arterial thrombosis. Mutations of the ITGA2 gene have been associated with vascular injury. It is therefore likely that a correlation exists between ITGA2 and KD. A meta-analysis suggested that ITGA2 rs1126643 affected susceptibility to aspirin insensitivity [10]. The average frequency of the ITGA2 gene rs1126643-T allele was 40.77% in Caucasian patients with aspirin insensitivity but was 58.58% in Chinese patients [10]. Here, we investigated whether ITGA2 SNP (rs1126643) polymorphism influences susceptibility to CAL in a cohort of 785 KD patients with or without CAL in the Chinese population.

## Materials and methods

### Subjects

A total of 300 patients with KD coronary artery lesions, diagnosed in line with the criteria of the American Heart Association in 2004 [11] who were receiving treatment in the Guangzhou Women and Children’s Medical Center, were enrolled. Additionally, 485 age- and sex-matched children patients with no-CAL(NCAL) were recruited in the same hospital, between February 2014 and February 2017. About 2 mL blood was collected from each participant for genomic DNA extraction (Qiagen, Dusseldorf, Germany). Informed consent was obtained from each participant’s guardian. This study was approved by the Institutional Committee of Guangzhou Women and Children’s Medical Center (2014073009).

### SNP selection and genotyping

Genomic DNA was extracted from peripheral blood samples for genotypic analysis of the SNPs (rs 11,226,643) of interest in the ITGA2 gene. Functional polymorphism was carried out based on a thorough evaluation of polymorphisms associated with vasculopathy [10, 11]. This was performed following methods reported previously [12]. Genomic DNA was extracted using the TIANamp Blood DNA Kit (Qiagen, Dusseldorf, Germany). The DNA samples were kept at − 80 °C until batch genotyping. Genotyping of the DNA was conducted via PCR using multiple gene-specific primer pairs targeting ITGA2 (rs1126643: forward GTGTTTAACTTGAACACATAT/ reverse AACTTG CATATTTTGCTT). The volume of the PCR mixture was 10 μl (2 × multiplex PCR mix + PCR primer pool and template DNA). The PCR protocol consisted of the following: 95 °C for 3 min; 15 cycles of 95 °C for 20s, 58 °C for 90s, and 72 °C for 30s; and 72 °C for 1 min using a GeneAmp PCR System 9700 (Thermo Fisher Scientific). The PCR products were subjected to massive parallel sequencing using an Ion Proton system (Life Technologies, CA, USA).

### Statistical analysis

The frequency distribution of the polymorphism and the demographic variables between KD with CAL and NCAL were compared using the χ^2^ test. The association of the ITGA2 rs1126643 C > T polymorphism with KD susceptibility was evaluated by calculating the odds ratio (OR) and 95% confidence interval (CI) using unconditional multivariate logistic regression analyses. Stratified analysis was based on coronary artery outcomes, age and sex, and *P*-values < 0.05 were considered statistically significant. Statistical analyses were performed using SAS software (Version 9.3; SAS Institute, Cary, NC, USA).

## Results

### Characteristics of the study population

The demographic characteristics of the KD patients with and without CAL are summarized in Table 1. The average age of patients was 28.48 months (±29.01, range = 1–166 months) for CAL and 31.23 months (±23.20, range = 1–110 months) for NCAL. There was no significant difference in age (*P* = 0.2411) or sex (*P* = 0.0603) between CAL and NCAL groups. Two-dimensional echocardiography revealed that 188 (62.67%) patients developed coronary artery dilatations/small aneurysms (SCAL), 63 (21.00%) developed coronary artery medium aneurysms (MCAL), and 49 (16.33%) developed giant coronary artery aneurysms (GCAL).

**Table 1:** Characteristics of KD cases with the CAL and NCAL group

Variables	CAL	NCAL	***P-***value^**a**^
Age range, month	1–166	1–110	
Mean ± SD	28.48 ± 29.01	31.23 ± 23.20	
≤60	271 (90.33%)	425 (87.63%)	0.2411
> 60	29 (9.67%)	60 (12.37%)	
sex			
Male	220 (73.33%)	325 (67.01%)	0.0603
Female	80 (26.67%)	160 (32.99%)	
Severity of CAL			
SCAL^b^	188 (62.67%)		
MCAL^c^	63 (21.00%)		
GCAL^**d**^	49 (16.33%)		

^a^ Two-sided χ2 test for distributions between Kawasaki disease patients with the CAL and NCAL. ^b^ Kawasaki disease patients with coronary artery dilatations/small aneurysms. ^c^ Kawasaki disease patients with coronary artery medium aneurysms. ^d^ Kawasaki disease patients with coronary artery giant aneurysms

### Association of ITGA2 gene polymorphism and KD with CAL susceptibility

The frequency distribution of the SNP polymorphism in the Kawasaki disease patients with and without CAL is shown in Table 2. The rs1126643 genotype showed increased risk of KD patients with CAL after adjustment for age and sex (CT vs CC: adjusted OR = 1.57, 95% CI = 1.16–2.12, *P* = 0.0032; CT/TT vs CC: adjusted OR = 1.49, 95% CI = 1.12–2.00, *P* = 0.0068; T vs C: adjusted OR = 1.66, 95% CI = 1.16–2.51, *P* = 0.0165). No other significant associations were detected. As a result, the harmful genotype used for the calculation was ITGA2 rs1126643 CT/TT.

**Table 2:** Genotype frequency distribution of the rs1126643 polymorphism in KD cases with the CAL and NCAL

Genotype	CAL (***n*** = 300)	NCAL (***n*** = 485)	***P-***value^**a**^	OR (95% CI)	***P-***value	Adjusted OR (95% CI)	Adjusted ***P-***value^**b**^
**ITGA2/rs1126643 C > T**							
CC	138 (46.00%)	273 (56.29%)		1.00		1.00	
CT	146 (48.67%)	182 (37.53%)		1.59 (1.18–2.14)	**0.0025**	1.57 (1.16–2.12)	**0.0032**
TT	16 (5.33%)	30 (6.19%)		1.06 (0.56–2.00)	0.8697	1.03 (0.54–1.95)	0.9374
Additive			**0.0089**	1.29 (1.02–1.64)	**0.0342**	1.28 (1.01–1.62)	**0.0443**
Dominant	162 (54.00%)	212 (43.71%)	**0.0050**	1.51 (1.13–2.02)	**0.0051**	1.49 (1.12–2.00)	**0.0068**
Recessive	284 (94.67%)	455 (93.81%)	0.6193	0.85 (0.46–1.60)	0.6216	0.83 (0.45–1.56)	0.5675
C	422 (70.33%)	728 (75.05%)	**0.0409**	1.00		1.00	
T	178 (29.67%)	242 (24.95%)		1.63 (1.08–2.47)	**0.0194**	1.66 (1.10–2.51)	**0.0165**

^a^ Two-sided χ2 test for distributions between Kawasaki disease patients with the CAL and NCAL. ^b^ Adjusted for age and sex status in logistic regress models

### Stratified analysis for polymorphism and Kawasaki disease susceptibility

The association of ITGA2 gene (rs1126643) polymorphism with KD coronary artery lesion susceptibility was further determined in stratified analysis by age, sex and severity of CAL (Table 3). Results showed that carriers of the CT/TT genotype had a significantly higher risk of KD coronary artery lesion especially for children ≤60 months of age (adjusted OR = 1.44, 95% CI = 1.06–1.96, *P* = 0.0199) and males (adjusted OR = 1.50, 95% CI = 1.06–2.11, *P* = 0.0218). The CT/TT genotype was significantly associated with an increased risk of SCAL (adjusted OR = 1.47, 95% CI = 1.04–2.08, *P* = 0.0277) and MCAL (adjusted OR = 1.72, 95% CI = 1.01–2.93, *P* = 0.0451) compared with carriers of the CC genotype.

**Table 3:** Stratification analysis of rs1126643 polymorphism in KD cases with the CAL and NCAL

Variables	rs1126643 (Cases/Controls)		***P***-value^**a**^	OR (95% CI)	***P***-value	Adjusted OR (95% CI)	Adjusted ***P***-value^**b**^
	CC	CT/TT					
Age, months							
≤ 60	126/238	145/187	**0.0143**	1.47 (1.08–1.99)	**0.0145**	1.44 (1.06–1.96)	**0.0199**
> 60	12/35	17/25	0.1328	1.98 (0.81–4.88)	0.1358	2.02 (0.82–4.99)	0.1292
sex							
Male	97/176	123/149	**0.0210**	1.50 (1.06–2.11)	**0.0214**	1.50 (1.06–2.11)	**0.0218**
Female	41/97	39/63	0.1670	1.47 (0.85–2.52)	0.1669	1.52 (0.88–2.61)	0.1357
Severity of CAL							
SCAL^c^	86/273	102/212	**0.0139**	1.53 (1.09–2.14)	**0.0142**	1.47 (1.04–2.08)	**0.0277**
MCAL^d^	27/273	36/212	**0.0444**	1.72 (1.01–2.92)	**0.0457**	1.72 (1.01–2.93)	**0.0451**
GCAL^**e**^	25/273	24/212	0.4803	1.24 (0.69–2.23)	0.4797	1.23 (0.68–2.22)	0.4992

^a^ Two-sided χ2 test for distributions between Kawasaki disease patients with the CAL and NCAL. ^b^ Adjusted for sex/age status in logistic regress models. ^c^ Kawasaki disease patients with coronary artery dilatations/small aneurysms. ^d^ Kawasaki disease patients with coronary artery medium aneurysms. ^e^ Kawasaki disease patients with coronary artery giant aneurysms

## Discussion

Here, we reveal an association of the ITGA2 gene (rs1126643) polymorphism with the risk of KD in 300 patients with CAL and 485 patients with NCAL. Specifically, the ITGA2 rs1126643 CT/TT was associated with an increased risk of KD patients with CAL, especially in children ≤60 months of age and males. Stratified analysis demonstrated that the ITGA2 rs1126643 CT/TT variant elevated the risk of KD progressing into SCAL and MCAL. To our knowledge, this is the first study to show that ITGA2 rs1126643 polymorphism is associated with KD coronary artery lesion susceptibility.

Integrins are adhesion molecules that promote platelet aggregation, hence the formation of blood clots [13]. ITGA2 is an important platelet receptor for collagen and regulates platelet activation by facilitating platelet adhesion and aggregation to the exposed surface of the subendothelial collagen fiber [14]. The gene encoding ITGA2 contains a number of polymorphisms, such as ischemic stroke and idiopathic sudden sensorineural hearing loss (iSSNHL) [15, 16]. ITGA2 has been implicated in thrombotic and arterial atherosclerotic disease. As a key member of the integrin family of adhesion molecules, ITGA2 mediates cell-cell, cell-matrix, and cell-matrix-cell adhesions [17]. It therefore affects various physiological and pathological processes such as inflammatory reactions, immune responses, atherosclerosis, and thrombosis [18]. KD is characterized by multisystem involvement and inflammation of all medium-sized arteries, including the coronary artery [19]. High infiltration of inflammatory cells in KD vascular tissues cause vascular damage during the acute febrile phase of KD [20]. In our study, we found that ITGA2 rs1126643 was a harmful factor with KD coronary artery lesions. To our knowledge, this is the first study to validate the association of ITGA2 rs1126643 with KD coronary artery lesions risk in a Chinese population. It may play a significant role in the pathogenesis of KD coronary artery lesions.

Although the etiology of KD is elusive, a genome-wide association study (GWAS) showed that single-nucleotide polymorphisms (SNPs) of AGT, NEBL, ITPKS, TGF-β, and KCNN2 may be modify the occurrence of CAL in KD [21–25]. To date, no study has explored the association of ITGA2 with KD coronary artery lesions. In the present study, we found that ITGA2 rs1126643 was associated with increased KD with coronary artery lesion susceptibility in the Chinese population. Stratified analysis showed that ITGA2 rs1126643 prevented the formation of SCAL and MCAL with KD.

Compared with the rs1126643 CC genotype, the harmful effect of the CT/TT variant genotype was more pronounced in children ≤60 months of age and in males. This finding is consistent with results of other studies [11, 26]. KD with CAL results in more apparent effects in young children below 60 months of age. The peak age of onset ranged from 9 to 11 months, and the male to female ratio was 1.5 to 1 [11, 26, 27]. Our results indicate that ITGA2 gene polymorphism is among the important factors affecting CAL in children with KD with CAL. Therefore, children with KD CAL may be predisposed to CAL and should be monitored to initiate early prevention and treatment. However, these results are based on a small sample size. In addition, we only collected ethnicity, geographical factors, age and sex as cases and controls. However, other ITGA2 gene polymorphisms were not involved in this study.

These results underscore the need to monitor the possibility of CAL in children with KD based on ITGA2 gene mutation. Early initiation of interventions will reduce the risk of mortality caused by CAL in children with KD.

## Conclusion

In summary, this study confirms that ITGA2 rs1126643 is associated with increased KD and coronary artery lesion susceptibility. However, future studies with larger sample sizes and practical experiments should be conducted to explore the roles of the ITGA2 gene in KD with coronary artery lesion susceptibility and severity.

## Data Availability

Data sharing is not applicable to this article, as no datasets were generated or analyzed during the current study. Please contact the author for data requests.

## Abbreviations

KD: Kawasaki disease
CAL: Coronary artery lesions
ITGA2: Integrin α2
SCAL: Kawasaki disease patients with coronary artery dilatations/small aneurysms
MCAL: Kawasaki disease patients with coronary artery medium aneurysms
GCAL: Kawasaki disease patients with coronary artery giant aneurysms
NCAL: No-CAL

## References

[1] Kawasaki T, Kosaki F, Okawa S, Shigematsu I, Yanagawa H (1974). A new infantile acute febrile mucocutaneous lymph node syndrome (MLNS) prevailing in Japan. Pediatrics..

[2] Huang WC, Huang LM, Chang IS, Chang LY, Chiang BL, Chen PJ (2009). Epidemiologic features of Kawasaki disease in Taiwan, 2003-2006. Pediatrics..

[3] Wang CL, Wu YT, Liu CA, Kuo HC, Yang KD (2005). Kawasaki disease: infection, immunity and genetics. Pediatr Infect Dis J.

[4] Kato H, Koike S, Yamamoto M, Ito Y, Yano E (1975). Coronary aneurysms in infants and young children with acute febrile mucocutaneous lymph node syndrome. J Pediatr.

[5] Kuo HC, Huang YH, Chien SC, Yu HR, Hsieh KS, Hsu YW (2014). Genetic variants of CD209 associated with Kawasaki disease susceptibility. PLoS One.

[6] Liang CD, Kuo HC, Yang KD, Wang CL, Ko SF (2009). Coronary artery fistula associated with Kawasaki disease. Am Heart J.

[7] Yim D, Curtis N, Cheung M, Burgner D (2013). Update on Kawasaki disease: epidemiology, aetiology and pathogenesis. J Pediatr Child Health.

[8] Kim HJ, Choi EH, Lim YJ, Kil HR (2017). The usefulness of plateleet-derived microparticle as biomarker of antipaltelet therapy in Kawasaki disease. J Korean Med Sci.

[9] Simon M, Kouskouni E, Vitoratos N, Economou E, Creatsas G (2017). Polymorphisms of platelet glycoprotein receptors and cell adhesion molecules in fetuses with fetal growth restriction and their mothers as detected with pyrosequencing. In Viv.

[10] Weng Z, Li X, Li Y, Lin J, Peng F, Niu W (2013). The association of four common polymorphisms from four candidate genes (COX-1, COX-2, ITGA2B, ITGA2) with aspirin insensitivity: a meta-analysis. PLoS One.

[11] Newburger JW, TakahashI M, Gerber MA, Gewitz MH, Tani LY, Burns JC (2004). Diagnosis, treatment , and long-term management of Kawasaki disease: a statement for health professionals from the committee on Rheumatic Fever, Endocarditis, and Kawasaki Disease, Council on Cardiovascular Disease in the Young, American Heart Association. Pediatrics.

[12] Che D, Pi L, Xu YF, Fu LY, Zhou HZ, Wang ZP (2018). TBXA2R rs4523 G allele is associated with decreased susceptibility to Kawasaki disease. Cytokine..

[13] Rivera J, Lozano ML, Navarro-Nú-ez L, Vicente V (2009). Platelet receptors and signaling in the dynamics of thrombus formation. Haematologica..

[14] Sixma JJ, van Zanten GH, Huizinga EG, van der Plas RM, Verkley M, Wu YP (1997). Platelet adhesion to collagen: an update. Thromb Haemost.

[15] Ballesteros F, Tassies D, Reverter JC, Alobid I, Bernal-Sprekelsen M (2012). Idiopathic sudden sensorineural hearing loss: classic cardiovascular and new genetic risk factors. Audiol Neurotol.

[16] Wu GL, Xi YJ, Yao L, Su L, Yan Y, Li MZ (2014). Genetic polymorphism of ITGA2 C807T can increase the risk of ischemic stroke. Int J Neurosci.

[17] Huo Y, Ley K (2001). Adhesion molecules and atherogenesis. Acta Physiol Scand.

[18] Lu JX, Lu ZQ, Zhang SL, Zhi J, Chen ZP, Wang WX (2014). Polymorphism in integrin ITGA2 is associated with ischemic stroke and altered serum cholesterol in Chinese individuals. Balkan Med J.

[19] Ahn JG, Bae Y, Shin D, Nam J, Kim KY, Kim DS (2019). HMGB1 gene polymorphism is associated with coronary artery lesions and intravenous immunoglobulin resistance in Kawasaki disease. Rheumatology(Oxford).

[20] Matsubara T, Ichiyama T, Furukawa S (2005). Immunological profile of peripheral blood lymphocytes and monocytes/macrophages in Kawasaki disease. Clin Exp Immunol.

[21] Kuo HC, Li SC, Guo MM, Huang YH, Yu HR, Huang FC (2016). Genome-wide association study identifies novel susceptibility genes associated with coronary artery aneurysm formation in Kawasaki disease. PLoS One.

[22] Zaitsu M, Hamasaki Y, Tashiro K, Matsuo M, Ichimaru T, Fujita I (2000). Ulinastatin, an elastase inhibitor, inhibits the increased mRNA expression of prostaglandin H2 synthase-type 2 in Kawasaki disease. J Infect Dis.

[23] Furie B, Furie BC (2004). Role of platelet P-selectin and microparticle PSGL-1 in thrombus formation. Trends Mol Med.

[24] Hildebrandt P (2009). Natriuretic peptides: prediction of cardiovascular disease in the general population and high risk populations. Dis Markers.

[25] Liu YF, Fu LY, Pi L, Che D, Xu YF, Zheng H (2019). An Angiotensinogen Gene Polymorphism (rs5050) Is Associated with the Risk of Coronary Artery Aneurysm in Southern Chinese Children with Kawasaki Disease. Dis Makers.

[26] Singh S, Vignesh P, Burgner D (2015). The epidemiology of Kawasaki disease: A global update. Arch Dis Child.

[27] Yanagawa H, Nakamura Y, Yashiro M, Uehara R, Oki I, Kayaba K (2006). Incidence of Kawasaki disease in Japan: the nationwide surveys of 1999–2002. Pediatr Int.

